# Exploitation of Tolerance of Wheat Kernel Weight and Shape-Related Traits from *Aegilops tauschii* under Heat and Combined Heat-Drought Stresses

**DOI:** 10.3390/ijms22041830

**Published:** 2021-02-12

**Authors:** Gamila Mohamed Idris Elhadi, Nasrein Mohamed Kamal, Yasir Serag Alnor Gorafi, Yuji Yamasaki, Kanenori Takata, Izzat S. A. Tahir, Michel O. Itam, Hiroyuki Tanaka, Hisashi Tsujimoto

**Affiliations:** 1 United Graduate School of Agricultural Sciences, Tottori University, Tottori 680-8553, Japan; j_mohamed@live.com (G.M.I.E.); itammichaelo@gmail.com (M.O.I.); 2 Arid Land Research Center, Tottori University, Tottori 680-0001, Japan; renokamal@tottori-u.ac.jp (N.M.K.); yasirserag@tottori-u.ac.jp (Y.S.A.G.); yujyamas@tottori-u.ac.jp (Y.Y.); 3 Wheat Research Program, Agricultural Research Corporation, P.O. Box 126, Wad Medani, Sudan; izzatahir@yahoo.com; 4 National Agriculture and Food Research Organization, Fukuyama 721-8514, Japan; ktakata@obihiro.ac.jp; 5 Faculty of Agriculture, Tottori University, Tottori 680-8550, Japan; htanaka@tottori-u.ac.jp

**Keywords:** *Aegilops tauschii*, heat stress, combined heat–drought stress, kernel weight, GWAS, grain yield

## Abstract

Kernel weight and shape-related traits are inherited stably and increase wheat yield. Narrow genetic diversity limits the progress of wheat breeding. Here, we evaluated kernel weight and shape-related traits and applied genome-wide association analysis to a panel of wheat multiple synthetic derivative (MSD) lines. The MSD lines harbored genomic fragments from *Aegilops tauschii*. These materials were grown under optimum conditions in Japan, as well as under heat and combined heat–drought conditions in Sudan. We aimed to explore useful QTLs for kernel weight and shape-related traits under stress conditions. These can be useful for enhancing yield under stress conditions. MSD lines possessed remarkable genetic variation for all traits under all conditions, and some lines showed better performance than the background parent Norin 61. We identified 82 marker trait associations (MTAs) under the three conditions; most of them originated from the D genome. All of the favorable alleles originated from *Ae. tauschii*. For the first time, we identified markers on chromosome 5D associated with a candidate gene encoding a RING-type E3 ubiquitin–protein ligase and expected to have a role in regulating wheat seed size. Our study provides important knowledge for the improvement of wheat yield under optimum and stress conditions. The results emphasize the importance of *Ae. tauschii* as a gene reservoir for wheat breeding.

## 1. Introduction

Abiotic stresses such as heat and drought affect yield negatively by reducing grain size and number. While the stresses in any growth stage affect the final yield, those at the grain filling stage are the most crucial [1,2]. Heat and drought stress in all developmental stages influence important morphological traits [3,4]. To adapt to these harsh environments, great efforts have been made to produce genetically tolerant plants and to understand the mechanisms behind the stress tolerance [5,6]. To identify genomic regions responsible for grain yield under stress conditions, analysis of the yield components is crucial [7]. Kernel weight is considered the most important and heritable trait among these components [8]. This trait is closely associated with kernel shape-related traits, such as kernel length, kernel width and kernel diameter [9]. The improvement of kernel weight and shape-related traits under stressed conditions is a promising approach to increasing wheat production [10]. In addition, screening for shape-related traits using image analysis can provide an easy and accurate means to assess yield components.

Wheat genetic diversity has been narrowed down in extensive breeding programs and thus, finding new genetic diversity in wild species is indispensable for improvement [11,12]. Many studies have used *Aegilops* species as genetic resources and reported their tolerance to abiotic stresses [13,14]. Among the related wild species, *Aegilops tauschii* is the most promising species because it has a D genome common with that of bread wheat and because no special cytological technique is needed to induce homologous recombination [15]. In this study, we employed wheat multiple synthetic derivative (MSD) lines, which contain the genetic diversity of a large accession of a wild species, *Aegilops tauschii*, and are suitable materials for genetic analysis [16].

Great progress has been made in identifying major QTLs for kernel size and shape [17,18,19,20,21,22], and several candidate genes were identified in wheat. For instance, cytokinin oxidase (encoded by *TaCKX*) reversibly inactivates cytokinin and increases kernel weight [23]. Cell wall invertase (encoded by *TaGW1*) regulates kernel size by sink tissue development [24], and RING-type E3 ubiquitin–protein ligase (encoded by *TaGW2*) increases kernel weight and size [25].

These studies describe kernel development under normal conditions, but extensive studies under stress conditions have not been conducted. To breed wheat genotypes that maintain grain yield even under stress conditions, knowledge of genotypic and environment interaction is necessary. Therefore, this study aimed at identifying the phenotypic and genotypic variation of kernel weight and shape-related traits among MSD lines under optimum (OP), heat (H) and combined heat–drought (HD) conditions, and to reveal the genetic mechanism of the productivity under stress, from the view point of kernel traits. Our results revealed a great diversity among the MSD lines in kernel weight and other kernel shape-related traits under all conditions. We identified promising markers and alleles that will contribute to our understanding of productivity under stressed condition and could be used in wheat breeding after validation.

## 2. Results

### 2.1. Phenotypic Variation of Kernel Weight and Shape-Related Traits under Optimum, Heat, and Combined Heat–Drought Conditions

**Figure 1:**
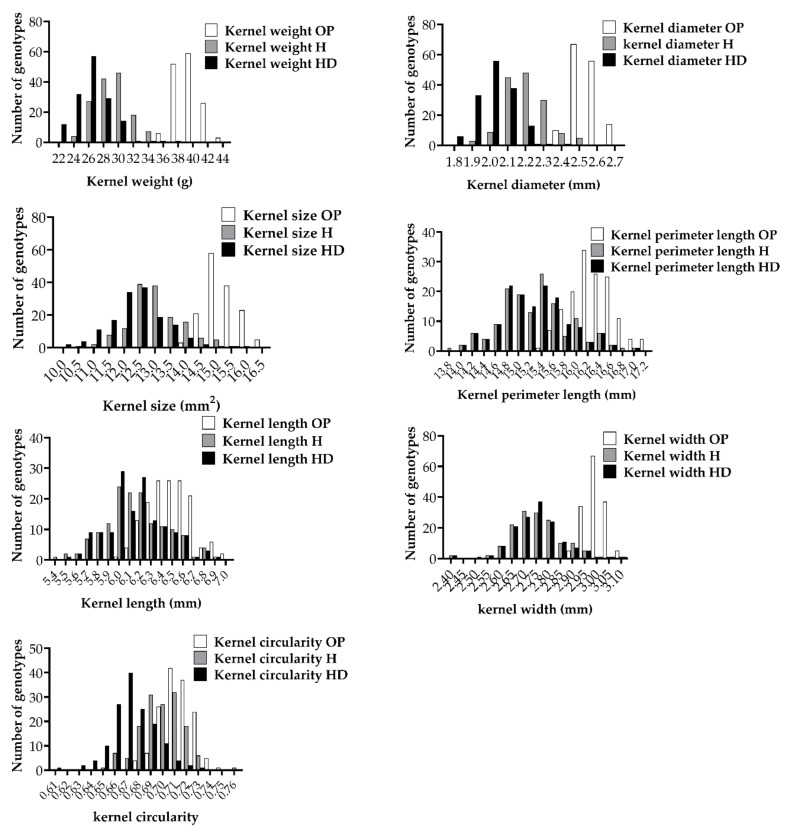
Frequency distribution of the multiple synthetic derivative lines grown under optimum (OP), heat (H) and combined heat–drought (HD) conditions. For the OP conditions, the predicted means of the values from two seasons, S1 and S2, were used.

MSD lines showed large phenotypic variation of all kernel traits under all three optimum (OP), heat (H) and combined heat–drought (HD) conditions (Figure 1). The MSD lines showed a wider range of variation under H and HD than under OP conditions, reflecting the MSD response to the stress conditions (Figure 1). The values of the kernel weight and shape traits were reduced under H and HD conditions, resulting in the means shifting towards the low values. The effects on kernel weight, kernel diameter, kernel size and kernel width were more potent than those on kernel length and kernel circularity (Figure 1).

Under OP conditions, the genotypic effect was significant for all traits except kernel width (Table 1). Differences were significant between the two seasons (S) in all traits except kernel length, and the interaction (G × S) was significant for all traits (Table 1). Under H and HD conditions, differences among genotypes were significant for all traits. The environment (E) affected all traits except kernel length, and G × E had no significant differences in any traits (Table 1). Moderate heritability (*H*^2^) (0.42 to 0.67) was observed under OP conditions for all traits except kernel width, which had a low heritability value (0.29). In contrast, high *H*^2^ was observed for all the traits under the stress environments (0.88 to 0.97) (Table 1).

**Figure 2:**
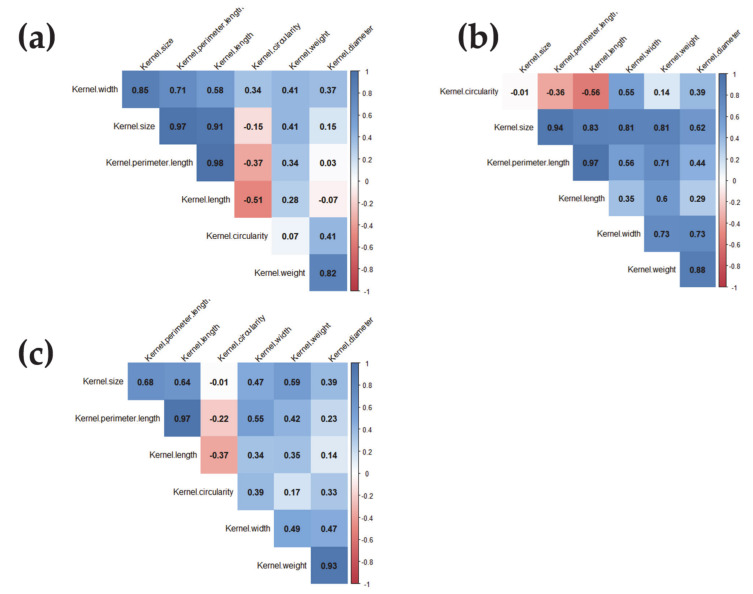
Correlation coefficient for kernel weight and shape-related traits under (**a**) optimum; (**b**) heat; and (**c**) combined heat–drought conditions. Absolute values >0.15 were significant at *p* = 0.05; >0.20 were significant at *p* = 0.01 under optimum, heat and combined heat–drought conditions, whereas absolute values > 26, 29 and 27 were significant at *p* = 0.001 under optimum, heat and combined heat–drought conditions, respectively.

All kernel traits except kernel circularity correlated positively with kernel weight under all conditions; the correlation with kernel diameter was the strongest (Figure 2). Correlations were positive among kernel size, kernel perimeter length and kernel length (Figure 2).

**Figure 3:**
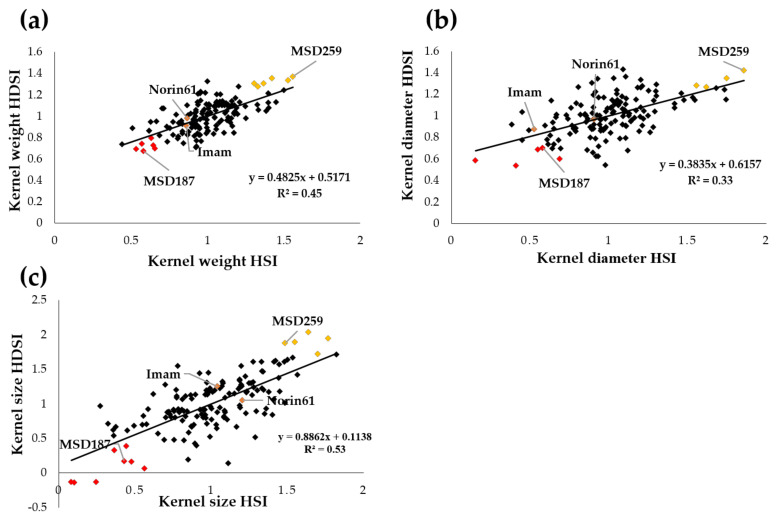
Regression analysis of the relationship between the heat susceptibility index (HSI) and the combined heat–drought susceptibility index (HDSI) for kernel (**a**) weight; (**b**) diameter; and (**c**) area size. Red color indicates tolerant lines with an index value of about 0.5 and yellow color indicates sensitive lines with an index value higher than one. The genetic background parent of the MSD lines Norin 61, the leading Sudanese cultivar Imam and two representative MSD lines showing high (MSD259) and low (MSD187) reductions are indicated.

To understand the performance of MSD lines under stress conditions (H and HD), we calculated the heat susceptibility index (HSI) and the combined heat–drought susceptibility index (HDSI) on the basis of the traits most strongly correlated with kernel weight, i.e., kernel diameter and kernel size. We performed a regression analysis between the HSI and HDSI to identify any relationship between the performances of MSD lines under the different stresses (Figure 3). There was an association between the MSD lines’ performance under H and HD conditions (*R*^2^ = 0.45, 0.33 and 0.53 for kernel weight, kernel diameter and kernel size, respectively). The HSI and HDSI of the background parent of the MSD lines Norin 61 and leading Sudanese cultivar Imam were around 1 (Figure 3), indicating that these genotypes showed levels of reduction similar to all genotypes studied, as seen in Figure 1.

**Figure 4:**
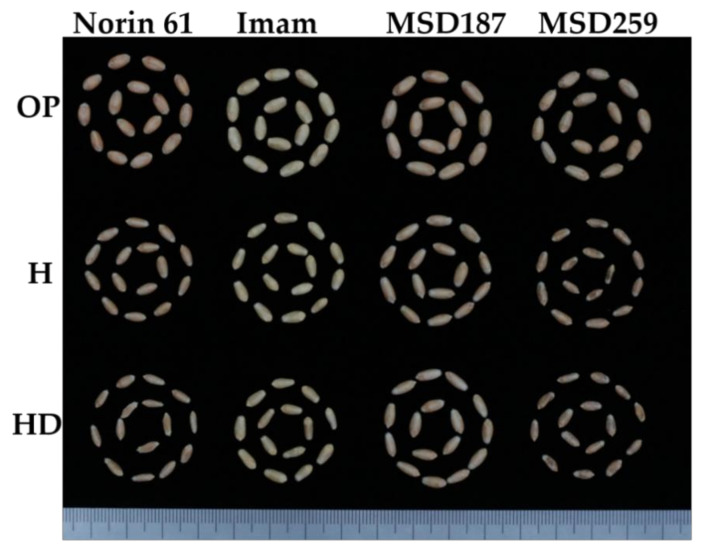
Variations in kernel weight and shape-related traits under optimum (OP), heat(H) and combined heat–drought (HD) conditions. Norin 61: the background of the MSD lines, showed a reduction under H and HD conditions; Imam: check cultivar known as heat tolerant, showed a small reduction; MSD187: showed a slight reduction under H and HD conditions; MSD259: highly sensitive line, showed remarkable reduction under H and HD conditions.

Interestingly, some MSD lines (indicated with red color) showed stable performance under stress conditions; they had the highest kernel weight, kernel diameter and kernel size under H and HD stress conditions (Figure 3). MSD187 performed well under all conditions in all three traits, whereas MSD259 was much more strongly affected by H and HD stresses than Norin 6” and Imam (Figure 3 and Figure 4).

### 2.2. Marker Trait Associations for Kernel Weight and Shape-Related Traits

We calculated the best linear unbiased predictions (BLUPs) for OP conditions with the data from two seasons, considering the significant seasonal effect in the association analysis. We identified 82 marker trait associations (MTAs) on different chromosomes under all conditions and for HSI and HDSI (Figures S1 and S2). Among the A, B and D genomes, the D genome had the highest MTA number under all conditions (Figure S3a).

**Figure 5:**
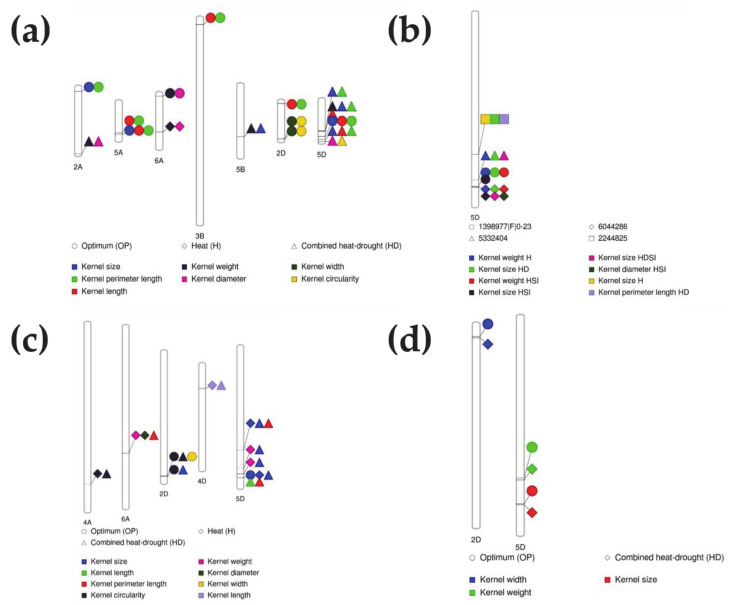
Markers for kernel weight and shape-related traits. (**a**) Pleiotropic markers under OP, H and HD conditions; (**b**) markers that showed potential for tolerance for kernel weight under H conditions and for kernel size under HD conditions and were associated with heat and heat–drought susceptibility indices; (**c**) stable markers between two or more environments; (**d**) markers that were located within distances less than 1 cM under different environments.

Under OP conditions, the A, B and D genomes contributed 30%, 13% and 57%, respectively (Figure S3b). Significant markers associated with kernel weight and kernel diameter were found on chromosome 6A and those associated with kernel weight, kernel size, kernel length and kernel perimeter length were found on chromosome 5D (Figure S4, Table 2). Significant markers on chromosome 3B were associated with kernel length, kernel perimeter length and kernel circularity, while those on 7B and 7D were associated with kernel width and those on 2B and 7A with kernel length (Figure S4, Table 2). Chromosome 2D had the highest number of MTAs, most of them associated with kernel circularity. The contribution (*R*^2^) of markers ranged from 0.074 in marker 1113863, associated with kernel diameter, on chromosome 2A to 0.16 in marker 1088563, associated with kernel circularity, on chromosome 2D (Table 2). Marker 5357358, on chromosome 2A, affected kernel size and kernel perimeter length (Figure 4, Table 2). In addition, markers 3028836 and 1088488|F|0-27, on chromosomes 5A and 5D, respectively, affected kernel size, kernel length and kernel perimeter length (Figure 5a, Table 2). Marker 4395641, on chromosome 6A, affected kernel diameter and kernel weight (Figure 5a, Table 2). In total, 30 MTAs were obtained under OP conditions (Table 2); some of them had pleiotropic effects, some were common between OP and HD conditions and others were associated with OP conditions (Table 2).

Under H conditions, 18 significant MTAs were detected (Figure S4, Table 2), i.e., about half of those detected under OP conditions (Table 2). All were located in the A (33%) and D genomes (67%), with no contribution from the B genome (Figure S3c). Chromosome 6A had markers associated with kernel weight, kernel diameter and kernel circularity, whereas chromosome 5D had markers associated with kernel weight and kernel size. Specific markers associated with kernel circularity were located on chromosomes 1A, 4A and 3D, and with kernel length on chromosome 4D (Figure S4, Table 2). *R*^2^ ranged from 0.08 in markers 987701, 1073897|F|0-27 and 5411945, associated with kernel diameter, kernel size and kernel circularity on chromosome 4A, 5D, and 6D respectively, to 0.13 in marker 1099241|F|0-17, associated with kernel diameter, on chromosome 6D (Table 2). No markers except 1073897|F|0-27, associated with kernel size, on chromosome 5D were detected under OP conditions, indicating their unique association with the kernel traits under H conditions. Marker 5968258, on chromosome 6A, affected kernel weight and kernel diameter (Figure 2a, Table 2).

Under HD conditions, we detected 43 MTAs (Figure S4, Table 2) with the highest contribution from the D genome (Figure S3d). Chromosome 5D had the highest number of MTAs that were associated with all kernel traits except kernel width; most of them were associated with kernel size (Table 2). We found MTAs associated with kernel size, kernel width and kernel circularity on chromosome 2D and with kernel length on chromosomes 3D and 4D (Figure S4, Table 2). In the B genome, significant MTAs for kernel size were found on chromosome 3B, and for kernel weight, kernel size and kernel circularity on chromosome 5B. In the A genome, MTAs for kernel weight, kernel diameter, kernel size and kernel width were found on chromosome 2A, for kernel circularity on chromosome 4A and for kernel size and kernel perimeter length on chromosome 6A (Figure S4, Table 2). *R*^2^ ranged from 0.08 for five markers to 0.19 in marker 3940004|F|0-21, associated with kernel width, on chromosomes 2D (Table 2) and 5A, respectively. Of the 43 MTAs, eight were also identified under H conditions, indicating their role in plant response to both stresses. On chromosome 2A, marker 1110845 affected kernel weight and kernel diameter, on chromosome 5B, marker 2248796|F|0-42 affected kernel weight and kernel size and on chromosome 5D, marker 991465 affected kernel weight, kernel size, kernel length and kernel perimeter length (Figure 2a, Table 2).

#### 2.2.1. MTAs for Heat and Combined Heat–Drought Susceptibility Indices

On the basis of HSI and HDSI (Table S1), 15 and 29 MTAs, respectively, were identified, of which the D genome contributed 61% (Table S1). Among them, marker 6044286, on chromosome 5D, was associated with the HSI for kernel weight and kernel diameter, and with both the HSI and HDSI for kernel size (Table S1). We also identified four associations for the HSI for kernel weight and kernel size on chromosome 5D, kernel weight on chromosome 2D and kernel diameter on chromosome 5D (Table S1). *R*^2^ ranged from 0.08 for marker 1019857|F|0-23, associated with the HSI for kernel diameter, to 0.15 for marker 1201315|F|0-67, associated with the HDSI for kernel diameter. Of 41 markers, five had pleiotropic effects (Table S1).

We identified four markers (1398977|F|0-23, 6044286, 5332404 and 2244825) on chromosome 5D under stress conditions (H and HD) that had pleiotropic effect for kernel weight and kernel size (Figure 5b). Those markers were associated with the HSI and HDSI of kernel weight and kernel size (Figure 5b)—for example, the marker 1398977|F|0-23, associated with the kernel size HDSI; 6044286, associated with the kernel size HSI, kernel weight HSI and kernel size HDSI; and 5332404, associated with the kernel size HDSI (Figure 5b).

#### 2.2.2. Stable MTAs for Kernel Weight and Shape-Related Traits

We identified nine stable markers under two or all three conditions, of which seven were on the D genome (Figure 5c). Among them, the marker 1073897|F|0-27 was associated with kernel size and stable under OP and H conditions and with kernel size, kernel perimeter length and kernel length under HD conditions (Figure 5c). Additionally, markers associated with kernel width on chromosome 2D and with kernel weight and kernel size on chromosome 5D were located close to each other (<1 cM) and were detected under OP and HD conditions (Figure 5d).

#### 2.2.3. Identification of Putative Candidate Genes for Kernel Weight and Shape-Related Traits

**Figure 6:**
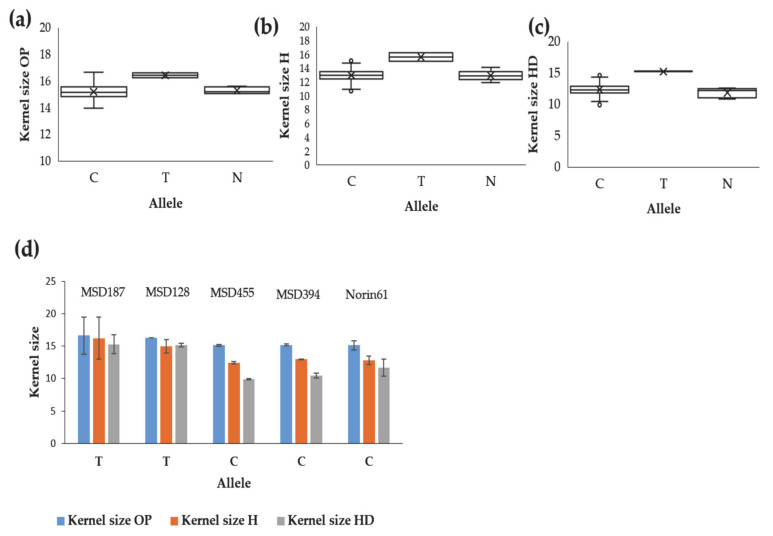
Effects of the alleles of the stable marker SNP_1073897|F|0-27 that increases kernel size under (**a**) optimum, (**b**) heat and (**c**) heat–drought conditions. (**d**) Examples of the lines harboring different alleles for SNP_1073897|F|0-27 and their parent Norin 61.

We searched for the candidate genes associated with the significant markers; we selected the candidate genes that possessed functions associated with kernel shape traits. The putative genes identified are listed in (Table 3). Under the three conditions, OP, H and HD, we identified genes related to stress tolerance, kernel size and yield regulation in the vicinity of the markers. Among 17 coding proteins in the region of the stable markers 1073897|F|0-27 associated with kernel size under OP, H, and HD conditions, we identified a putative RING-type E3 ubiquitin–protein ligase involved in kernel size and yield regulation and encoded by TraesCS5D02G504400 (Table 3). The introduced SNP including T contributed to increasing the kernel area size under OP, H and HD conditions. This SNP originated from the *Ae. tauschii* accessions KU-2155 and KU-2156 collected in Iran (Figure 6a–c). The lines MSD187 and MSD128 harboring this allele had higher kernel area sizes than their parent Norin 61 harboring the C allele under all conditions (Figure 6d).

**Figure 7:**
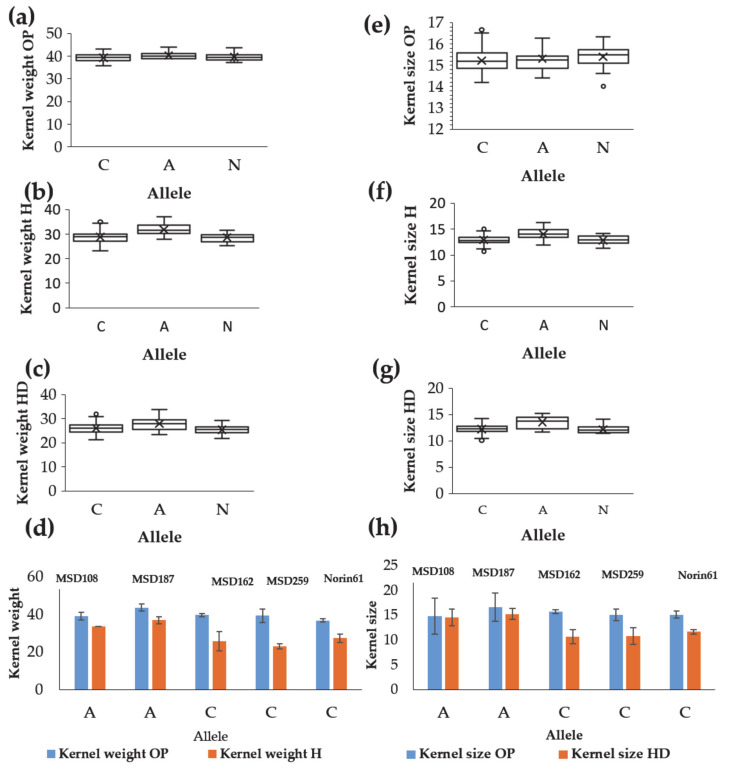
Effects of the alleles of marker 5332404, associated with the kernel weight under heat conditions (H), kernel size under combined heat–drought (HD) conditions and kernel size HD susceptibility index. (**a**–**c**) Effects on kernel weight under optimum (OP), H and HD conditions; (**e**–**g**) Effects on kernel size under OP, H and HD conditions. (**d**,**h**) Effects on kernel weight and kernel size of some MSD lines and Norin 61.

The marker 5332404 on chromosome 5D was associated with kernel weight under H conditions, kernel size under HD conditions and the kernel size HDSI (Table 2 and Table S1). There were nine coding proteins adjacent to this marker, among them the gene TraesCS5D02G445100 which encodes for a heat stress transcription factor associated with high-temperature stress tolerance (Table 3). The allele A of this marker originated from eight *Ae. tauschii* accessions (IG 126387, KU-2039, KU-2124, AT 80, KU-20-10, KU-2155, KU-2156 and PI 499262) and increased kernel weight and kernel size under H and HD but not under OP conditions (Figure 7e–g), indicating a special role for this allele under stress. Under stress, the MSD lines harboring the A allele had higher kernel weights and kernel sizes than Norin 61 or MSD lines with the C allele (Figure 7d,h).

## 3. Discussion

Seed shape and size are the most important agronomic traits owing to their effect on grain kernel weight. Few QTLs associated with kernel traits have been identified under stress conditions in wheat through the association mapping approach. Here, we aimed to detect the effects of heat and heat–drought on kernel weight and shape-related traits using a panel of unique MSD lines harboring different D genome sources.

### 3.1. Phenotypic Variation for Kernel Weight and Shape-Related Traits under Optimum and Stress Conditions

The responses to stress conditions were varied for the kernel traits, in which HD conditions severely affecting weight and shape-related traits, followed by the H conditions. We observed that kernel weight, kernel diameter and kernel size were the traits most affected by HD compared to H conditions. In addition, Norin 61, the backcross parent of the MSD lines, and the standard check cultivar Imam showed remarkable reductions in kernel weight and shape-related traits under HD compared to H conditions. Combined heat–drought severely affects plants due to heat stress evapotranspiration leading to severe drought stress [26]. Ramya et al. [27] reported that drought and heat stress shorten the grain growth period and lead to improper grain filling, thereby reducing the kernel weight and the overall yield. Moreover, high temperature reduces the conversion of sucrose to starch due to the suppression of the soluble starch synthase enzyme, leading to shriveled kernels [28]. Drought and heat stress accelerate leaf senescence, decrease photosystem II efficiency. As a result, this leads to the reduction of the amount of stored assimilates translocated into developing grains and reduced kernel size [29,30]. Heat and drought affect plant growth and thereby reduce yield [31]. In this study, kernel weight was severely affected by HD compared to H conditions. Qasem et al. [32] also observed that combined heat–drought significantly affect kernel weight and yield. Furthermore, shape-related traits, especially kernel diameter and size, were affected by HD compared to H conditions. However, kernel length was less affected by the stresses than kernel weight and other related traits (Table 1). This finding is in agreement with a report that kernel length is less affected by heat than the kernel weight [33]. We identified some MSD lines, such as MSD187, that could maintain good kernel weight under stress, unlike Norin 61 and Imam. These germplasm lines could be used in wheat breeding programs for heat stress tolerance [34].

Most of the kernel weight and shape traits, including kernel diameter and kernel weight, exhibited moderate heritability under OP conditions, whereas high heritability under H and HD conditions was found (Table 1). Xin et al. [35] found high heritability of kernel shape traits in four different environments. Traits with high heritability and genetic advances can be selected directly for crop improvement [36].

The association between kernel weight, kernel diameter, kernel size, kernel width and kernel length under all conditions indicated that all these traits contribute to kernel weight, as reported in [21,37] (Figure 2). Kernel diameter was most strongly correlated with kernel weight, with heritability ranging from 0.64 under OP conditions to 0.88 under stress. These results suggest kernel diameter as a target trait for selection in breeding programs aiming at increasing kernel weight and yield in wheat.

### 3.2. Marker Trait Associations for Kernel Weight and Shape-Related Traits under Optimum and Stress Conditions

More than 50% of the MTAs identified in this study were on the D genome, thus indicating its higher contribution, especially under stress conditions. This result is inconsistent with previous reports for the kernel shape that indicated a lower contribution of the D genome compared to A and B genomes [20,38,39]. Rasheed et al. [17] also reported a lower contribution of the D genome, though they used synthetic hexaploid wheat. This inconsistency could be attributed to the previous studies being conducted under non-stress conditions and/or to the diversity of the D genome in the materials used being narrower than the A and B genomes; our results are in agreement with Ali et al. [40]. On the other hand, our findings reveal the uniqueness of the MSD panel as an effective and powerful platform for allele and gene mining in *Ae. tauschii*.

Thousand-kernel weight is one of the yield components and QTL studies have been conducted for this trait [4]. Also, studies have identified QTLs for grain size at different chromosomes. However, further studies are necessary to understand these traits under stressed environments [41]. Here, in a genome-wide association study (GWAS) for kernel size, we identified a high peak on the chromosome 5D under the HD environment. Similar results were described by Afzal et al. [42], specifically, that chromosome 5D influences drought tolerance, indicating that this locus could have an important role in enhancing kernel size and yield under HD conditions. We identified markers associated with kernel weight on chromosomes 6A and 5B under H and HD conditions, respectively. Lopez et al. [41] reported similar results under H and HD conditions for kernel weight. Under all conditions, the identified MTAs indicated that kernel diameter was most strongly associated with kernel weight. Several studies have reported the association of kernel diameter with kernel weight and other traits under stress condition [43]. These findings further support the above phenotypic correlation findings that kernel diameter, besides other traits, can be a target for selection in breeding programs to increase kernel weight and final grain yield.

Some markers had pleiotropic effects. We found a putative gene, *TraesCS6A02G057300*, for one of these markers, 4395641, on chromosomes 6A, associated with kernel weight and kernel diameter. This gene encodes an F-box domain-containing protein (Table 3). F-box proteins regulate leaf senescence, flower development and defense responses [44,45]. They also have a role in ethylene signaling [46]. Reduction in ethylene signaling has been suggested to increase grain yield in maize and Arabidopsis [47], consistent with the critical role of kernel weight and kernel diameter in increasing wheat grain yield.

The candidate gene for the pleiotropic marker 2248796|F|0-42 for kernel weight and kernel size detected under HD conditions on chromosome 5B encodes aspartic proteinase nepenthesin-1. Yao et al. [48] found that the overexpression of an aspartic proteinase can play a role in drought avoidance through ABA signaling. A recent study in wheat reported that an aspartic proteinase is associated with wheat stress response [49].

Among pleiotropic markers on chromosome 5D detected under H and HD conditions, 5332404 had the candidate gene *TraesCS5D02G445100*. This gene encodes a heat stress transcription factor, which has an important role in responses to abiotic stresses [50]. The lines MSD187 and MSD108, harboring the positive allele of this marker, performed better than Norin 61 and the check cultivar Imam, indicating the usefulness of this marker in maintaining kernel weight under stress. After validation, this marker could be very useful in wheat breeding.

Marker 1076033|F|0-62, associated with kernel width under HD conditions, on chromosome 2D had the candidate gene *TraesCS2D02G076500*, which encodes the heat shock protein Hsp20 (Table 3). Heat shock proteins enhance plant immunity [51]. Recently, *TaHSP20* genes have been shown to play an important role in abiotic stress tolerance in wheat [52]. Therefore, this marker and its candidate gene could be used in marker-assisted selection programs to improve wheat stress tolerance.

In agreement with Ali et al. [40], we found that kernel area size was most closely associated with kernel perimeter length and then kernel length under all conditions. The candidate gene *TraesCS2A02G099400* for marker 5357358 detected under OP conditions on chromosome 2A encodes a basic leucine zipper transcription factor (Table 3). Basic leucine zipper is a member of the transcription factor families that controls transcription of seed maturation genes and is expressed during seed development [53].

We identified markers stable under at least two conditions. Marker 1073897|F|0-27 was associated with kernel size under all three conditions and is a good candidate for marker-assisted selection in breeding programs. Interestingly, the candidate gene for this marker, *TraesCS5D02G504400*, encodes a RING-type E3 ubiquitin–protein ligase, like *TaGW2* which increases grain size and yield [25]. The ubiquitin pathway plays a crucial role in determining plant seed size [54]. Wheat has *TaGW2* copies on chromosomes 6A, 6B and 6D. Here, we identified a stable marker and candidate gene under OP, H and HD conditions on chromosome 5D. This study used a panel of MSD derivatives with high diversity of the D genome derived from several *Ae. tauschii* accessions; we speculate that the marker identified in our study could be related to a new gene affecting seed size in wheat. However, a detailed study is necessary to confirm this assumption.

## 4. Materials and Methods

### 4.1. Plant Materials

This study used 400 BC_1_F_7_ multiple synthetic derivative lines developed by crossing and backcrossing the bread wheat (*T. aestivum*) cultivar Norin 61 and 43 synthetic wheat lines [16]. The synthetic wheat lines were developed by crossing 43 different *Ae. tauschii* accessions with the durum wheat (*Triticum turgidum* ssp. *durum*) cultivar Langdon. We evaluated 400 lines under optimum conditions in Japan and 140 selected lines under stress conditions in Sudan. These 140 lines do not require vernalization treatment and are adapted to Sudanese conditions.

### 4.2. Field Experiments

Optimum conditions in Japan: the 400 MSD lines were grown in a field of the Arid Land Research Center (35°32′ N, 134°13′ E, 11 m asl), Tottori, Japan, in two seasons: 2015/16 and 2018/19. The soil was sandy (95% sand, 1.3% silt, 3.7% clay) [55]. Before sowing, three commercial fertilizer mixtures—Kumiai Fukugo PKN 366 (MC Ferticom Co., Ltd. Tokyo. Japan; 60 kg), Hitachi Fukugo 1 (Hitachi-kakou Co. Hitachi, Ibaraki, Japan, Ltd.; 40 kg) and granular carbonated magnesium lime (Shimizu Kogyo Co., Ltd. Tokyo, Japan; 100 kg)—were spread onto the soil. During the tillering stage, Kodo Kasei 444 (Mitsubishi Syoji Agri-service Co., Ltd. Osaka, Japan; 50,000 kg ha^−1^) was spread. The experiment was arranged in an augmented randomized complete block design with eight blocks. We used four replicated checks with Norin 61 (the MSD parent), Imam and Tagana (Sudanese heat-tolerant cultivars) and Safedak Ishkashim (a Tajikistan landrace) in each block. Each line was grown in a row of five plants with 0.2 m between plants. The seeds were sown in late October and plants were harvested in mid-June. The average temperature was 11.9 °C in the 2015/16 and 11.5 °C in the 2018/19 seasons. The minimum/maximum temperatures were −3.8/26.2 °C in 2015/16 and 1.8/25.3 °C in 2018/19; the average temperatures during maturity (May–June) were 20.1 °C and 19.7 °C, respectively.

Heat (H) and heat–drought (HD) conditions: the lines were grown at the Gezira Research Farm (GRF), Agricultural Research Corporation (ARC), Wad Medani, Sudan (14°24′ N, 29°33′ E, 407 m asl), in the 2017/18 season from November to March. We selected GRF because it is recognized as the global center for heat-tolerance research [56,57]. The ARC manages it in collaboration with CIMMYT, ICARDA and Tottori University, Japan (SATREPS Project). This farm is within a clay plain and the soil is heavy clay Vertisol (pH 8.5). Before sowing, P was applied at 18.8 kg ha^−1^. Seeds were treated with Gaucho insecticide (imidacloprid, 35% WP, Bayer Crop Science) at 1 g kg^−1^ seeds and sown at 120 kg ha^−1^ manually in the fourth week of November in an alpha lattice design, with two replications. Plot size was four 1.0-m rows with 0.2 m between rows. In H plots, plants were irrigated every 10–12 days, as recommended by the ARC. In HD plots, water supply was withheld when 50% of the lines reached anthesis. Data loggers (Em50, Decagon Devices, Pullman, WA, USA) connected to sensors (Terso21, Decagon Devices) were used to measure soil water potential at 20 cm depth and the plants were re-watered when the potential reached −900 kPa to avoid permanent wilting stress. Nitrogen was applied twice as urea at the three-leaf stage (second irrigation) and at the tillering stage (fourth irrigation) at 86 kg ha^−1^.

### 4.3. Measurement of Kernel Weight and Shape-Related Traits

From each MSD line in each plot and each replication in all environments, 100 grains were used for kernel trait measurements. Kernel weight and kernel diameter were measured using a single-kernel characterization system (SKCS 4100, Perten Instruments) at the National Agriculture Research Center for Western Region, Fukuyama, Hiroshima, Japan. Kernel shape parameters (area size, perimeter length, length, width, and circularity) were analyzed in SmartGrain software v. 1.2 [58] with up to 100 intact seeds. Circularity was calculated by an equation, 4π (area size)/(perimeter length)^2^.

### 4.4. Statistical Analysis of Kernel Weight and Shape-Related Traits

Analysis of variance (ANOVA) for an augmented randomized complete block design was performed using Plant Breeding Tools software (PBTools, v. 1.4, International Rice Research Institute, Laguna, Philippines http://bbi.irri.org/products (accessed on 9 February 2021)). GenStat 18 (VSN International, Rothamsted Research, Harpenden, Hertfordshire, UK) was used to carry out the ANOVA for alpha lattice design experiments (H and HD conditions). Broad-sense heritability (*H*^2^) was calculated using PBTools. Pearson’s correlation coefficients were performed using R software with a custom script in the ggcorplot package (R core team 2021), available at http://www.sthda.com/english/wiki/ggcorrplot-visualization-of-a-correlation-matrix-using-ggplot2 (accessed on 9 February 2021). Heat susceptibility indices under H (HSI) and HD (HDSI) conditions were calculated for kernel weight, kernel size and kernel diameter as:

*HSI (or HDSI) = (1 − Yh/Y)/(1 − Xh/X),* (1)

where *Yh* is the phenotypic mean of each genotype under H or HD conditions; *Y* is the phenotypic mean of each genotype under OP conditions; *Xh* is the mean of all lines under H or HD conditions; and *X* is the mean of all lines under OP conditions.

### 4.5. Genome-Wide Association Study and Candidate Gene Identification

The MSD lines and Norin 61 were genotyped using the DArT-seq platform (Diversity Arrays Technology, Bruce, Australia https://www.diversityarrays.com (accessed on 9 February 2021)) [16]. GWAS for kernel weight and shape-related traits was performed with 14,355 DArT-seq markers in TASSEL5 v. 20,151,113 software [59]. We used a mixed linear model (MLM) with PCA and a kinship matrix to account for population structure and cryptic relationships. Manhattan plots were generated using −log_10_ (*P*). The adjusted *P* < 3 × 10^−3^ was used as a threshold to determine significant association. To identify the candidate genes, significant markers sequences were used for the search in Unité de Recherche Génomique Info, Versailles, France (URGI: https://urgi.versailles.inra.fr/ (accessed on 9 February 2021)), with the blast option used for comparisons with the International Wheat Genome Sequencing Consortium, Castanet Tolosan Cedent, France (WGSC) RefSeq V.1 chromosomes. We searched for the candidate genes 0.5 Mb upstream and downstream of the positions of the significant markers.

## 5. Conclusions

In this study, we examined an MSD population with broad diversity in the D genome of bread wheat. The MSD lines were remarkably variable in the kernel traits under OP, H and HD conditions. We identified many MTAs, most of which were on the D genome, revealing the power of the MSD lines as a platform for gene mining in *Ae. tauschii*. Some MSD lines performed better than the backcross parent Norin 61 and the check cultivar Imam under H and HD stress conditions. These lines, along with the stable markers, favorable alleles and candidate genes elucidated here, represent a good resource to enhance wheat grain yield under stress and optimum conditions. However, more work will be necessary to validate the suitability of these markers and/or alleles. Nevertheless, our study supports the claim that *Ae. tauschii* is an important gene reservoir to breed stress-resilient bread wheat.

## Figures and Tables

**Table 1:** Analysis of variance and heritability of kernel weight and shape-related traits under optimum (OP), heat (H) and combined heat–drought (HD) conditions for multiple synthetic derivatives lines.

	**Kernel Weight (g)**			**Kernel Diameter (mm)**			**Kernel Size (mm^2^)**		
	SED±	*H* ^2^	*p*-value	SED±	*H* ^2^	*p*-value	SED±	*H* ^2^	*p*-value
C (OP)	3.33	–	***	0.13	–	***	1.94		**
S (S1 × S2)	–	–	*	–	–	***	–	–	**
G × S	3.39	0.56	***	0.13	0.64	***	1.94	0.42	**
H	1.53	–	***	0.08	–	***	0.60	–	***
HD	1.73	–	***	0.09	–	***	0.61	–	***
E	–	–	***	–	–	***	–	–	***
G × E	1.75	0.92	ns	0.07	0.88	ns	0.55	0.94	ns
	**Kernel Length (mm)**			**Kernel Width (mm)**			**Kernel Circularity**		
	SED±	*H* ^2^	*p*-value	SED±	*H* ^2^	*p*-value	SED±	*H* ^2^	*p*-value
C (OP)	0.46	–	***	0.22		ns	0.02	–	***
S (S1 × S2)	–	–	ns	–	–	***	–	–	**
G × S	0.46	0.51	***	0.22	0.29	*	0.02	0.67	**
H	0.13	–	***	0.07	–	***	0.01	–	***
HD	0.18	–	***	0.08	–	***	0.01	–	***
E	–	–	ns	–	–	***	–	–	***
G × E	0.14	0.97	ns	0.07	0.88	ns	0.01	0.92	ns

C: combined analysis for the two seasons under OP conditions; SED±: standard error of differences; ns, not significant; * *p* < 0.05, ** *p* < 0.01, *** *p* < 0.001.

**Table 2:** Marker trait associations of kernel weight and shape-related traits in multiple synthetic derivative lines grown under optimum (OP), heat (H) and combined heat–drought (HD) conditions.

Chromosome	Marker Position (Bp)	Marker	Trait	Conditions	*p*-Value	*R* ^2^
1A	229673410	1115856|F|0-14	Kernel circularity	H	9.75 × 10^−4^	0.10
2A	1696501	3026123|F|0-8	Kernel weight	HD	2.40 × 10^−4^	0.10
	17036238	5357358	Kernel size	OP	2.49 × 10^−4^	0.12
			Kernel perimeter length	OP	7.51 × 10^−4^	0.10
	57562452	3938604	Kernel width	HD	1.08 × 10^−3^	0.09
	149553891	1113863	Kernel diameter	OP	1.67 × 10^−3^	0.07
	250645685	1110845	Kernel diameter	HD	9.00 × 10^−4^	0.08
			Kernel weight	HD	7.72 × 10^−4^	0.08
	244243147	5010926	Kernel size	HD	1.02 × 10^−3^	0.08
3A	39514910	1035262|F|0-46	Kernel circularity	HD	8.80 × 10^−5^	0.11
4A	184463076	987701	Kernel circularity	H	1.01 × 10^−3^	0.08
				HD	8.28 × 10^−4^	0.09
5A	117490096	1079158	kernel length	OP	5.02 × 10^−4^	0.10
			Kernel perimeter length	OP	5.47 × 10^−4^	0.10
	117719229	1157204|F|0-51	Kernel perimeter length	OP	9.12 × 10^−4^	0.10
	118132341	977527|F|0-25	Kernel perimeter length	OP	9.13 × 10^−4^	0.10
	124441377	3028836	Kernel size	OP	5.09 × 10^−4^	0.10
			Kernel length	OP	6.30 × 10^−4^	0.09
			Kernel perimeter length	OP	3.78 × 10^−4^	0.10
	127107886	3940004|F|0-21	Kernel width	HD	1.11 × 10^−4^	0.19
6A	945226	2277231	Kernel circularity	H	7.23 × 10^−4^	0.09
	10617494	4395641	Kernel weight	OP	2.94 × 10^−4^	0.10
			Kernel diameter	OP	1.67 × 10^−3^	0.08
	12184855	1115036	Kernel weight	OP	5.48 × 10^−4^	0.09
	15813131	4407451	Kernel weight	OP	1.99 × 10^−4^	0.10
	32701094	1019857|F|0-23	Kernel diameter	H	2.03 × 10^−4^	0.10
	77372430	7327852	Kernel size	HD	4.35 × 10^−4^	0.09
	86323044	3945281	Kernel diameter	H	4.39 × 10^−4^	0.09
	133562282	3384829	Kernel size	HD	6.62 × 10^−4^	0.09
	145087238	5968258	Kernel weight	H	7.75 × 10^−4^	0.09
			Kernel diameter	H	5.89 × 10^−4^	0.10
			Kernel perimeter length	HD	9.15 × 10^−4^	0.08
7A	160041813	1056001	Kernel length	OP	9.34 × 10^−4^	0.10
2B	118229752	7351021|F|0-10	Kernel length	OP	5.20 × 10^−4^	0.09
3B	24450611	3958195	Kernel length	OP	1.21 × 10^−4^	0.12
			Kernel perimeter length	OP	3.86 × 10^−4^	0.10
	678386865	4396161	Kernel circularity	OP	9.64 × 10^−4^	0.08
	755958103	7353565	Kernel size	HD	8.84 × 10^−4^	0.09
5B	109565358	1302570	Kernel circularity	HD	4.34 × 10^−4^	0.11
	196141955	2248796|F|0-42	Kernel weight	HD	5.07 × 10^−4^	0.11
			Kernel size	HD	2.91 × 10^−4^	0.12
6B	48765954	3020427	Kernel size	HD	5.31 × 10^−4^	0.11
	191946279	993061	Kernel circularity	HD	6.60 × 10^−4^	0.10
7B	205492257	5581272	Kernel size	HD	1.80 × 10^−4^	0.11
	240968931	3949081	Kernel width	OP	6.80 × 10^−4^	0.10
	246168466	4260892	Kernel width	OP	7.13 × 10^−4^	0.10
2D	9318714	4999702	Kernel width	OP	9.34 × 10^−4^	0.08
	10253062	1000563|F|0-24	Kernel length	OP	1.00 × 10^−4^	0.14
			Kernel perimeter length	OP	3.28 × 10^−4^	0.12
	142248333	3946155	Kernel circularity	OP	2.54 × 10^−4^	0.14
			Kernel circularity	HD	2.48 × 10^−4^	0.11
			Kernel width	OP	2.20 × 10^−4^	0.11
	142982233	3957018	Kernel circularity	OP	7.92 × 10^−5^	0.14
			Kernel size	HD	7.55 × 10^−4^	0.09
	143867561	4542702	Kernel circularity	OP	1.25 × 10^−4^	0.14
			Kernel circularity	OP	3.95 × 10^−4^	0.14
	10453150	1076033|F|0-62	Kernel width	HD	0.00107	0.08
	143981582	3534099	Kernel width	OP	6.51 × 10^−4^	0.09
	147520289	1088563	Kernel circularity	OP	3.21 × 10^−5^	0.16
	147746144	4992154|F|0-9	Kernel circularity	OP	3.14 × 10^−4^	0.15
	149031034	1116168|F|0-15	Kernel circularity	OP	2.72 × 10^−4^	0.13
	149318699	3023828	Kernel circularity	OP	4.58 × 10^−4^	0.12
3D	762269	3385313|F|0-6	Kernel length	HD	8.41 × 10^−4^	0.10
	112899800	1203228|F|0-45	Kernel circularity	H	1.01 × 10^−3^	0.11
	113047617	1122898|F|0-41	Kernel circularity	H	4.03 × 10^−4^	0.13
4D	27756435	1001438|F|0-46	Kernel length	H	1.09 × 10^−3^	0.10
			Kernel length	HD	8.93 × 10^−4^	0.10
5D	59337192	1099271|F|0-23	Kernel weight	HD	9.63 × 10^−4^	0.10
	112083542	1091823|F|0-41	Kernel weight	H	9.80 × 10^−4^	0.10
	117708268	1072444	Kernel size	HD	8.30 × 10^−4^	0.09
	118171820	2244825	Kernel size	H	6.65 × 10^−4^	0.09
			Kernel size	HD	1.09 × 10^−3^	0.09
			Kernel perimeter length	HD	5.62 × 10^−4^	0.09
	119535444	991465	Kernel weight	HD	4.95 × 10^−4^	0.09
			Kernel size	HD	1.39 × 10^−5^	0.15
			Kernel length	HD	7.49 × 10^−4^	0.09
			Kernel perimeter length	HD	7.08 × 10^−4^	0.09
	120017850	1201315|F|0-67	Kernel diameter	HD	4.98 × 10^−4^	0.13
	120914450	7351647|F|0-44	Kernel size	HD	4.90 × 10^−4^	0.11
	121111673	4910927	Kernel weight	OP	1.05 × 10^−3^	0.08
	138730779	1088488|F|0-27	Kernel size	OP	2.82 × 10^−4^	0.13
			Kernel length	OP	3.89 × 10^−4^	0.12
			Kernel perimeter length	OP	1.69 × 10^−4^	0.13
	139543900	5332404	Kernel weight	H	8.05 × 10^−4^	0.09
			Kernel size	HD	2.38 × 10^−4^	0.11
	140397428	3955838|F|0-28	Kernel size	HD	6.41 × 10^−4^	0.12
	140551286	1101952	Kernel size	HD	2.07 × 10^−4^	0.12
	140719410	7350532	Kernel size	HD	5.78 × 10^−4^	0.09
	141061760	1087740	Kernel size	HD	2.32 × 10^−4^	0.11
	141600686	1215969	Kernel size	HD	6.05 × 10^−5^	0.12
	142135700	3941995	Kernel size	HD	4.32 × 10^−4^	0.13
	142429952	3946915	Kernel size	HD	1.08 × 10^−3^	0.09
	142680984	3954584	Kernel size	HD	6.87 × 10^−5^	0.13
	143115886	3026564	Kernel size	HD	7.86 × 10^−5^	0.13
	143728332	6041628	Kernel size	HD	6.01 × 10^−5^	0.12
	144829140	1398977|F|0-23	Kernel weight	H	6.45 × 10^−4^	0.11
			Kernel size	HD	6.21 × 10^−5^	0.17
	145561848	6044286	Kernel weight	H	3.88 × 10^−4^	0.11
	145561848		Kernel size	HD	1.64 × 10^−5^	0.17
	145561848	1696241	Kernel size	HD	5.62 × 10^−4^	0.09
	148482930	2257612|F|0-47	Kernel size	HD	4.10 × 10^−4^	0.09
	150113559	1073897|F|0-27	Kernel size	OP	8.53 × 10^−4^	0.08
			Kernel size	H	8.24 × 10^−4^	0.08
			Kernel size	HD	2.54 × 10^−4^	0.09
			Kernel length	HD	5.84 × 10^−4^	0.08
			Kernel perimeter length	HD	3.56 × 10^−4^	0.09
	151218934	1218899|F|0-6	Kernel size	HD	1.10 × 10^−4^	0.14
	158302411	1041586|F|0-42	Kernel diameter	HD	7.44 × 10^−4^	0.10
			Kernel circularity	HD	4.98 × 10^−4^	0.13
6D	895318	5411945	Kernel diameter	H	1.06 × 10^−3^	0.08
	15112276	1099241|F|0-17	Kernel diameter	H	2.01 × 10^−4^	0.13
	22055487	1019857|F|0-52	Kernel diameter	H	2.05 × 10^−4^	0.10
	176474631	1268158	Kernel circularity	HD	5.33 × 10^−4^	0.09
7D	196638923	5331548	Kernel width	OP	1.05 × 10^−3^	0.08

**Table 3:** Candidate genes for kernel weight and shape-related traits under optimum, heat and combined heat–drought conditions and their putative physiological roles.

Marker	Chromosome	Trait (Environment)	*R*^2^	Gene	Protein	Function
5357358	2A	Kernel size (OP), kernel perimeter length(OP)	0.11	*TraesCS2A02G099400*	Basic-leucine zipper (bZIP) transcription factor family protein	Regulates seed maturation
4407451	6A	Kernel weight (OP)	0.10	*TraesCS6A02G056600*	Auxin-responsive protein	Regulates auxin
4395641	6A	Kernel weight (OP), kernel diameter (OP)	0.10	*TraesCS6A02G057300*	F-box domain-containing protein	Flower development, defense response
2248796|F|0-42	5B	Kernel weight (HD), kernel size (HD)	0.11	*TraesCS5B02G302400*	Aspartic proteinase nepenthesin-1	Role in drought avoidance
1076033|F|0-62	2D	Kernel width (HD)	0.08	*TraesCS2D02G076500*	Heat shock protein Hsp20 domain-containing protein	Tolerance to biotic and abiotic stresses
5332404	5D	Kernel weight (H), kernel size (HD)	0.09	*TraesCS5D02G445100*	Heat stress transcription factor	Tolerance to environmental stress
1073897|F|0-27	5D	Kernel size (OP, H, HD), kernel length (HD), kernel perimeter length (OP, HD)	0.08	*TraesCS5D02G504400*	E3 ubiquitin–protein ligase	Regulates grain size and yield
1398977|F|0-23	5D	Kernel weight (H)	0.11	*TraesCS5D02G469900*	F-box domain-containing protein	Flower development, defense response

## Data Availability

Not applicable.

## Supplementary Materials

Supplementary Materials can be found at https://www.mdpi.com/1422-0067/22/4/1830/s1. Figure S1. Manhattan plots for kernel weight and shape-related traits under optimum (OP), heat (H) and combined heat–drought (HD) conditions. (a) Manhattan plot for kernel weight; (b) Manhattan plot for kernel size; (c) Manhattan plot for kernel diameter; (d) Manhattan plot for kernel perimeter length; (e) Manhattan plot for kernel length; (f) Manhattan plot for kernel width; (g) Manhattan plot for kernel circularity. Figure S2. Manhattan plot for heat susceptibility index (HSI) and heat drought susceptibility index (HDSI) for (a) kernel weight; (b) kernel diameter; and (c) kernel size. Figure S3. Percent contribution of marker trait associations (MTAs) in the A, B and D genomes of bread wheat. (a) All MTAs identified; (b) MTAs identified under OP conditions; (c) MTAs under H conditions; and (d) MTAs under HD conditions. Figure S4. Significant marker trait associations of kernel weight and shape traits under optimum, heat and combined heat–drought conditions. Table S1. Markere trait associations of heat suscibtability index and combined heat drought suscibtability index of kernel weight and shape-related traits in multiple synthetic derivatives lines grown under optimum, heat and combined heat drought conditions.

## Abbreviations

ANOVA	Analysis of variance
BLUP	Best linear unbiased prediction
GWAS	Genome-wide association study
H	Heat
HD	heat–drought
HDSI	Heat–drought susceptibility index
HSI	Heat susceptibility index
MSD	Multiple synthetic derivatives
MTA	Marker trait association
OP	Optimum
