# Supplementary figures and images for: Exploitation of Tolerance of Wheat Kernel Weight and Shape-Related Traits from Aegilops tauschii under Heat and Combined Heat-Drought Stresses
Source: Int J Mol Sci. 2021 Feb 12;22(4):1830. doi: 10.3390/ijms22041830 (PMC7917938; doi:10.3390/ijms22041830)

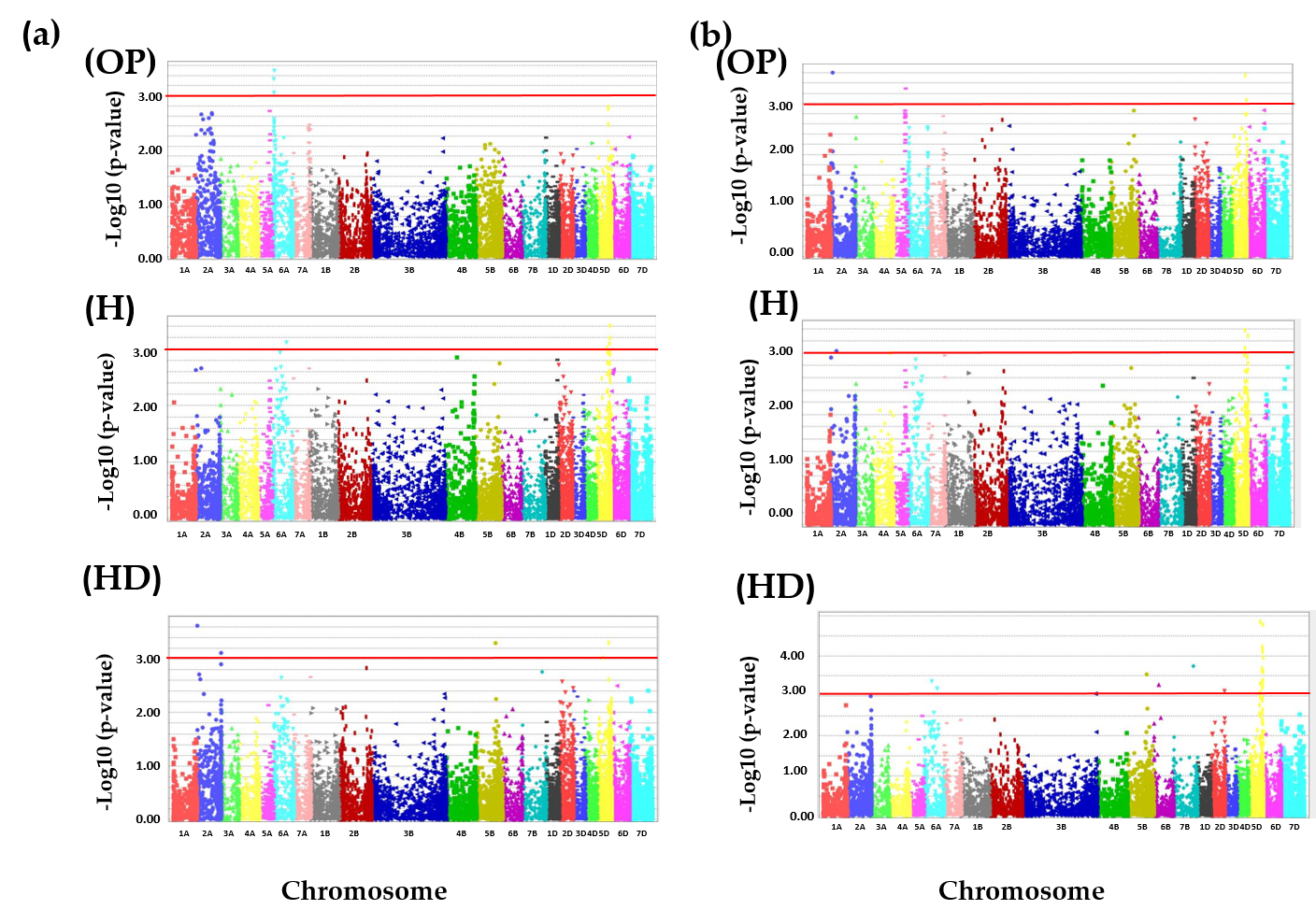

Supplement: Supplementary file 1 [file ijms-22-01830-s001.zip › Figure S1-1.tif]

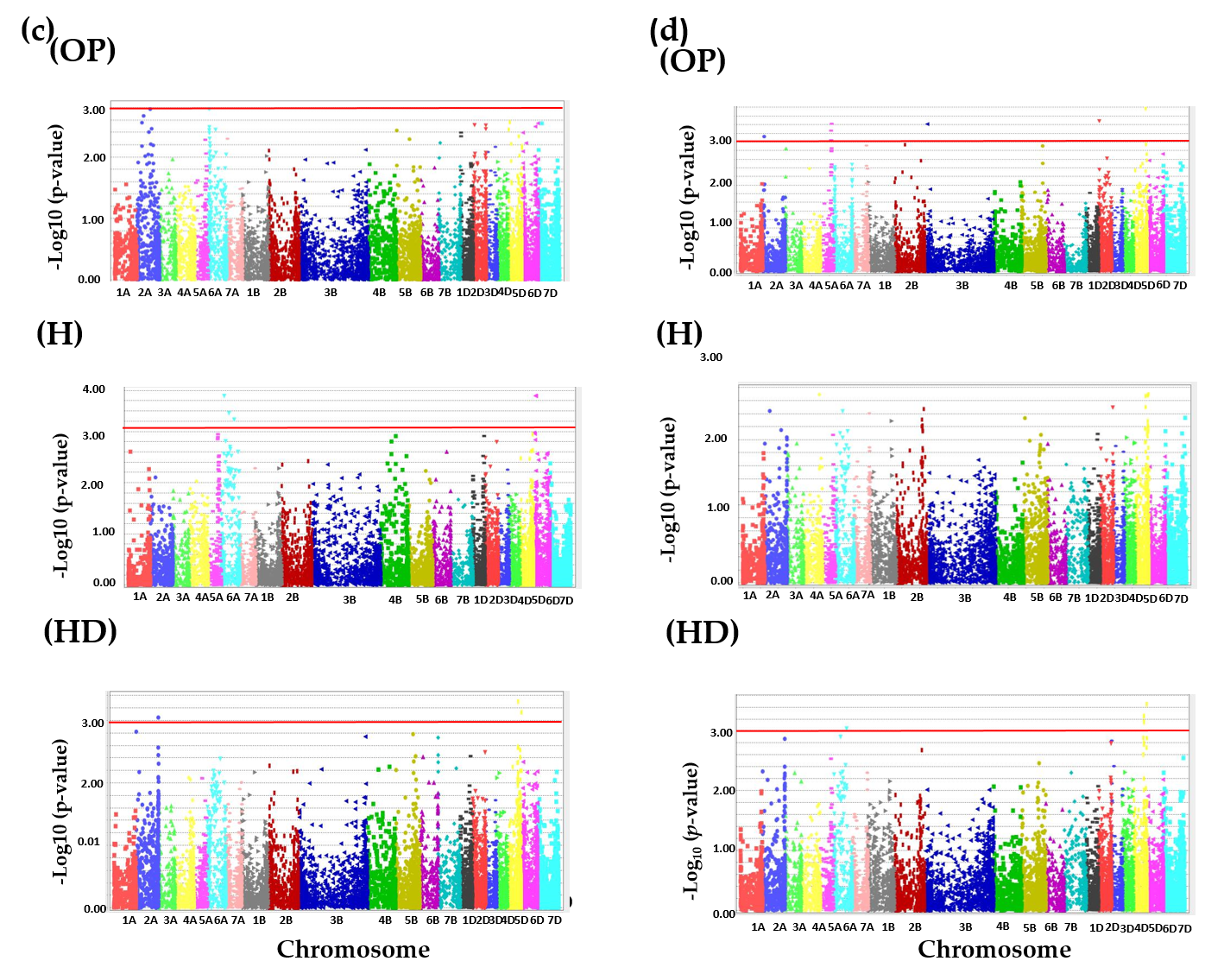

Supplement: Supplementary file 1 [file ijms-22-01830-s001.zip › Figure S1-2.tif]

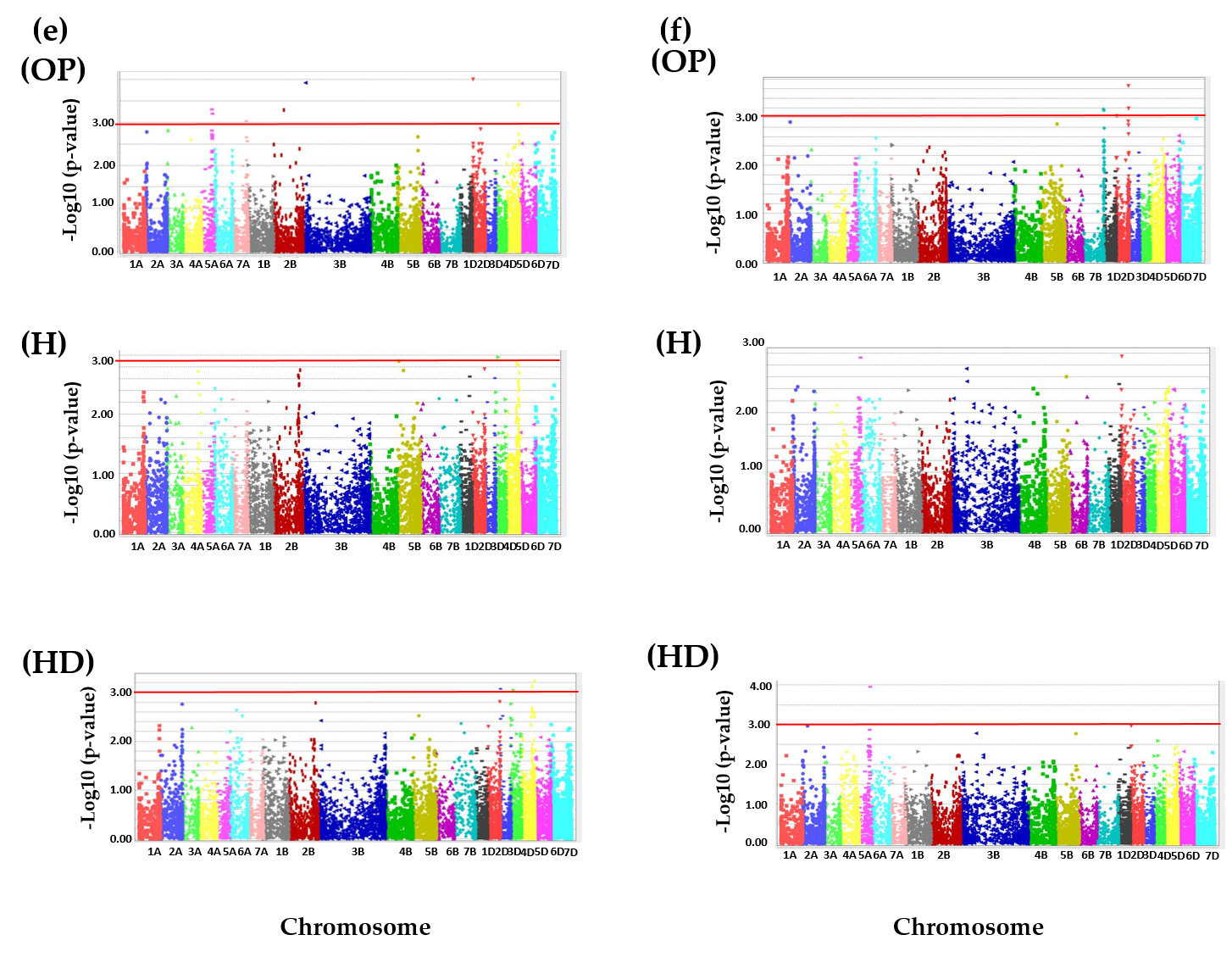

Supplement: Supplementary file 1 [file ijms-22-01830-s001.zip › Figure S1-3.tif]

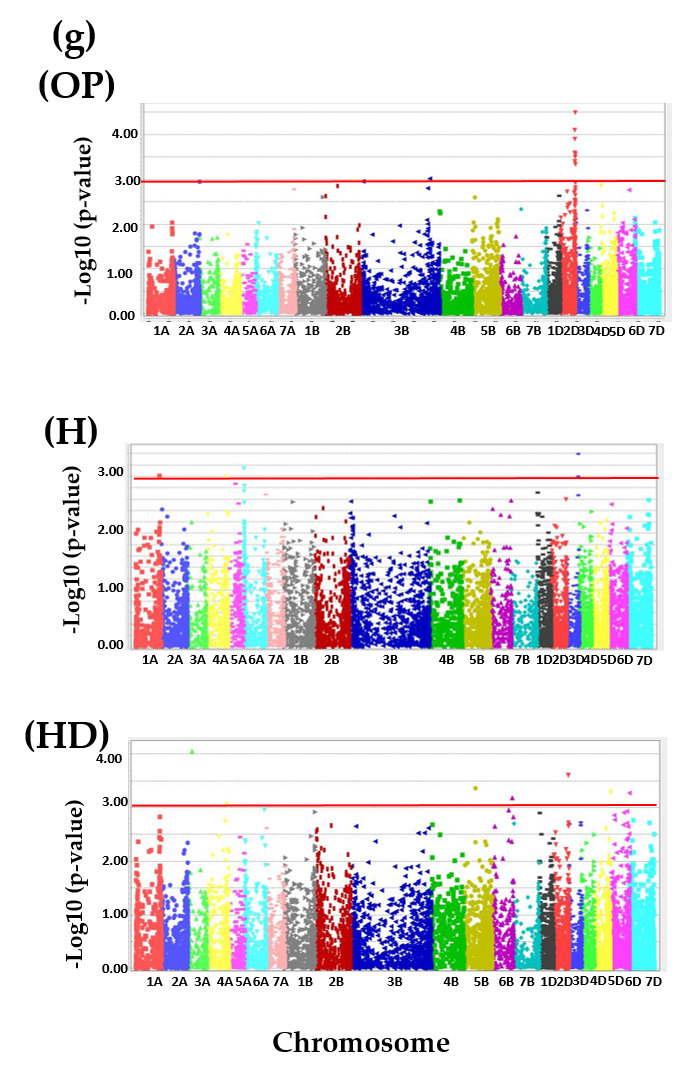

Supplement: Supplementary file 1 [file ijms-22-01830-s001.zip › Figure S1-4.tif]

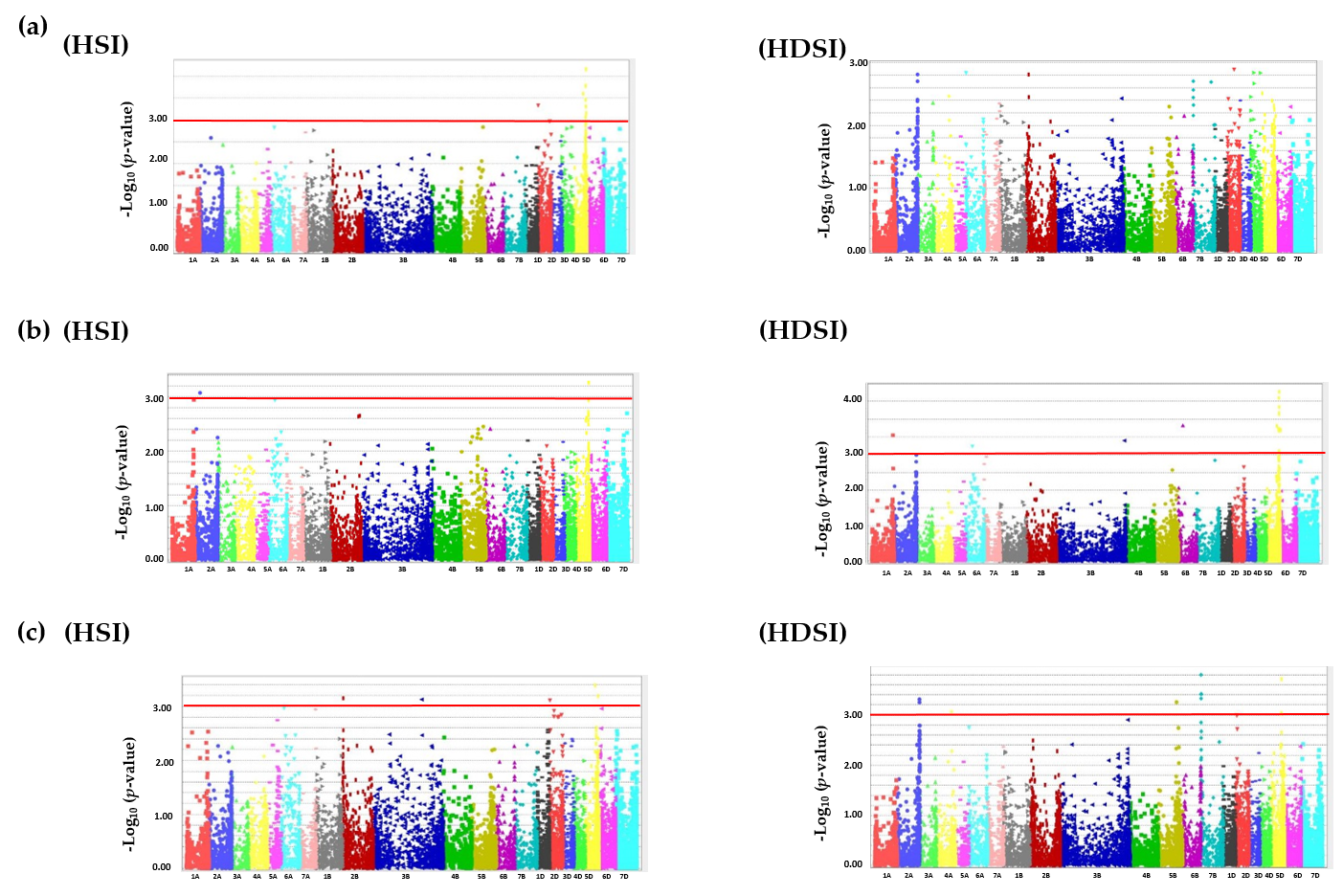

Supplement: Supplementary file 1 [file ijms-22-01830-s001.zip › Figure S2.tif]

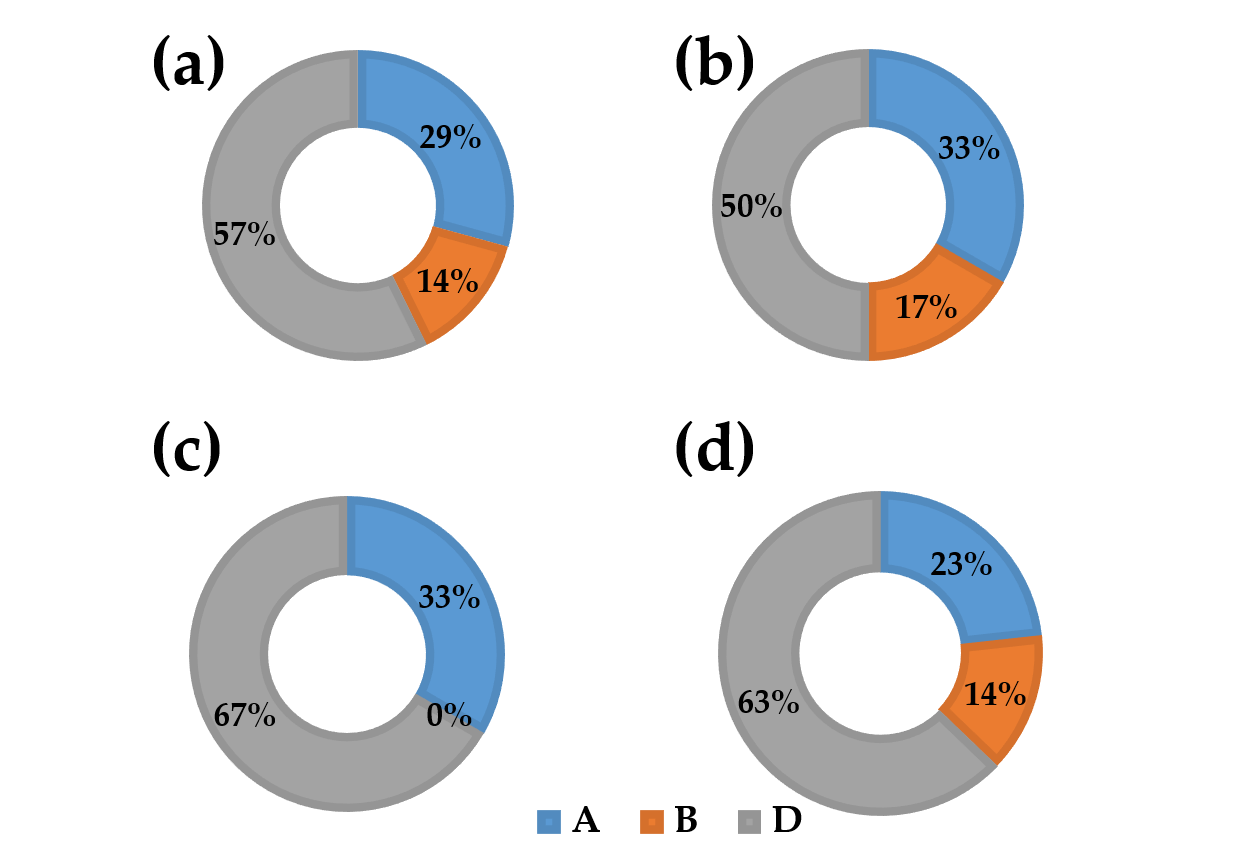

Supplement: Supplementary file 1 [file ijms-22-01830-s001.zip › Figure S3.tif]
